# FDG-PET/CT Limited to the Thorax and Upper Abdomen for Staging and Management of Lung Cancer

**DOI:** 10.1371/journal.pone.0160539

**Published:** 2016-08-24

**Authors:** Anne I. J. Arens, Jan W. A. Postema, Wendy M. J. Schreurs, Albert Lafeber, Baudewijn W. Hendrickx, Wim J. G. Oyen, Wouter V. Vogel

**Affiliations:** 1 Department of Nuclear Medicine, Dr. Bernard Verbeeten Institute, Tilburg, The Netherlands; 2 Department of Nuclear Medicine, Radboud university medical center, Nijmegen, The Netherlands; 3 Department of Nuclear Medicine, Atrium Medical Centre, Heerlen, The Netherlands; 4 Department of Nuclear Medicine, The Netherlands Cancer Institute—Antoni van Leeuwenhoek Hospital, Amsterdam, The Netherlands; 5 Department of Nuclear Medicine, Rijnstate Hospital, Arnhem, The Netherlands; Universite de Bretagne Occidentale, FRANCE

## Abstract

**Purpose:**

This study evaluates the diagnostic accuracy of [F-18]-fluorodeoxyglucose-positron emission tomography/computed tomography (FDG-PET/CT) of the chest/upper abdomen compared to the generally performed scan from head to upper thighs, for staging and management of (suspected) lung cancer in patients with no history of malignancy or complaints outside the thorax.

**Methods:**

FDG-PET/CT scans of 1059 patients with suspected or recently proven lung cancer, with no history of malignancy or complaints outside the thorax, were analysed in a retrospective multi-centre trial. Suspect FDG-avid lesions in the chest and upper abdomen, the head and neck area above the shoulder line and in the abdomen and pelvis below the caudal tip of the liver were noted. The impact of lesions detected in the head and neck area and abdomen and pelvis on additional diagnostic procedures, staging and treatment decisions was evaluated.

**Results:**

The head and neck area revealed additional suspect lesions in 7.2%, and the abdomen and pelvis in 15.8% of patients. Imaging of the head and neck area and the abdomen and pelvic area showed additional lesions in 19.5%, inducing additional diagnostic procedures in 7.8%. This resulted in discovery of additional lesions considered malignant in 10.7%, changing patient management for lung cancer in 1.2%. In (suspected) lung cancer, PET/CT limited to the chest and upper abdomen resulted in correct staging in 98.7% of patients, which led to the identical management as full field of view PET in 98.8% of patients.

**Conclusion:**

High value of FDG-PET/CT for staging and correct patient management is already achieved with chest and upper abdomen. Findings in head and neck area and abdomen and pelvis generally induce investigations with limited or no impact on staging and treatment of NSCLC, and can be interpreted accordingly.

## Introduction

Lung cancer is diagnosed in an estimated 386.300 patients annually in Europe and in an estimated 1.8 million patients worldwide in 2012, and will continue to be a significant epidemic in the coming decades [1,2]. Prognosis is generally poor as most patients present with an advanced stage of the disease, with an overall 5-year survival of only 15% [3]. Therapeutic strategies with curative intent are available for selected subgroups [4]. Early detection and accurate evaluation of disease extent are essential to ensure appropriate treatment.

Detection and staging of lung cancer generally involves staging modalities such as, chest X-ray, CT-scans, bronchoscopy, echo-endoscopy, sonograhy of the abdomen, mediastinoscopy, PET-scan, each having a specific value which all can contribute to the staging [5,6]. To achieve quick and accurate staging at an acceptable cost and patient burden, multiple diagnostic procedures need to be combined in an optimal strategy.

Imaging for detection and staging of lung cancer is traditionally based on computed tomography (CT) of the thorax [7]. In many cases the diagnostic algorithm is extended with “whole body” positron emission tomography using [F-18]-fluorodeoxyglucose (FDG-PET), for non-invasive staging based on metabolic tissue characterization [8]. FDG-PET is integrated into clinical management as it has proven to be highly sensitive and specific for detection of lymph node metastases and distant metastases and it characterizes indeterminate lesions detected on CT [9–12]. CT and FDG-PET provide additive diagnostic information, nowadays acquired in a single session with integrated PET/CT scanners [13–15]. But PET/CT has not replaced CT for staging lung cancer.

CT for detection and staging of lung cancer has been standardized to fully include the structures of the thorax (ie thoracic inlet and inferior pleural reflections) which generally comprises a scanning field of view from the lower neck to the upper abdomen. This field of view includes the site of the primary tumour, possible locations of lymph node metastases (e.g. the lung hilus, mediastinum and supraclavicular regions) and many sites prone to distant metastases (e.g. the contralateral lung, the adrenal glands, a significant part of the liver and a large proportion of the axial skeleton) [16]. Sometimes an additional CT or MRI scan of the brain is performed to assess brain metastases. Body parts with a low diagnostic yield for this specific clinical application are generally not imaged with CT, such as the lower abdomen and pelvis, the arms, and the legs.

In contrast, FDG-PET for the detection and staging of lung cancer is traditionally performed in a “whole body” setting, although the exact scan field of view varies per institute, e.g. full body (head to toe) or only the central body (base of the skull to the upper thighs). It is well known that FDG-PET may reveal unexpected distant metastases or second primary tumours [11,17,18]. On the other hand, the rate of false-positive PET lesions in the head/neck and lower abdomen/pelvis may be relatively high, resulting in undesired treatment delays and patient stress due to additional investigations.

In this study we investigated the diagnostic value of FDG-PET/CT for the detection and primary staging of lung cancer, with and without body areas with a low pre-test likelihood of metastatic lesions.

## Materials and Methods

### Patients

All patients who were referred for lung cancer evaluation between September 2006 and September 2007 in 5 participating centers were eligible for inclusion (Table 1). Inclusion criteria were: suspected or recently proven lung cancer (either NSCLC or SCLC), referred for FDG-PET/CT imaging for tumour detection or staging, no history of prior malignant disease, and no signs or symptoms of malignant disease outside the thoracic area. Prior diagnostic imaging with other modalities (e.g. CT, bone scan) was not an exclusion criterion, as long as no distant metastases were suspected prior to FDG-PET/CT.

**Table 1:** Institutes and imaging protocols.

Participants		PET imaging					CT imaging
Institute	Location	PET/CT device	Scan Field of view	FDG dose (MBq)	Scan time (min/bp)	Reconstruction parameters	Scan parameters
BVI	Tilburg	Siemens Biograph 2 GE discovery STE16	Skull top-groins Skull top-groins	4.2/kg 4.2/kg	3.00 3.00	OSEM 2i 8s OSEM 2i 20s	Lowdose 30 mAs Lowdose 40 mAs
Atrium MC	Heerlen	Philips Gemini	Skull top-groins	3.7/kg	3.00–5.00	OSEM 2i 8s	CE Diagnostic 100 mAs
Radboud university medical center	Nijmegen	Siemens Biograph 2	Skull base-groins	3.5/kg	4.00	OSEM 2i 8s	Lowdose 40 mAs
Rijnstate Hospital	Arnhem	Philips Gemini	Skull top-groins	3.125/kg	1.45	LOR-RAMLA	Lowdose 40 mAs
NKI-AvL hospital	Amsterdam	Philips Gemini TOF	Skull base-groins	3/kg	2.15	TOF default	Lowdose 40 mAs

TOF = time of flight. CE = Enhanced with intravenous contrast.

The study has been carried out in the Netherlands in accordance with the applicable rules concerning the review of research ethics committees and informed consent.

We received a waiver of approval from the CMO region of Arnhem-Nijmegen. Consent was not needed to be obtained, as this was a retrospective study with anonymously analyzed data. The data was collected by AIJA, JWAP, WMJS, AL, BWH and WVV. The authors anonymized the data themselves. Some of the authors might have had interaction with some of the patients discussed within the study but not in context of the study, this interaction would only be the administration of FDG prior to the scan.

### FDG-PET/CT imaging

Scanner types, scan field of views and imaging protocols in the participating institutes are listed in Table 1. Local imaging protocols for the detection and staging of lung cancer were respected, as they reflect the current clinical practice in the Netherlands. A minimum scan field of view from the skull base to the upper thighs was required for FDG-PET/CT. FDG-PET/CT was performed after at least 4 hours of fasting. Diabetes mellitus needed to be regulated with fasting glucose levels < 11 mmol/l (<200 mg/dl), with no insulin administration shortly prior to FDG administration. Some centers performed low-dose CT, whilst one institution performed diagnostic quality contrast-enhanced CT of the whole scanning field of view.

### Image evaluation

**Fig 1:**
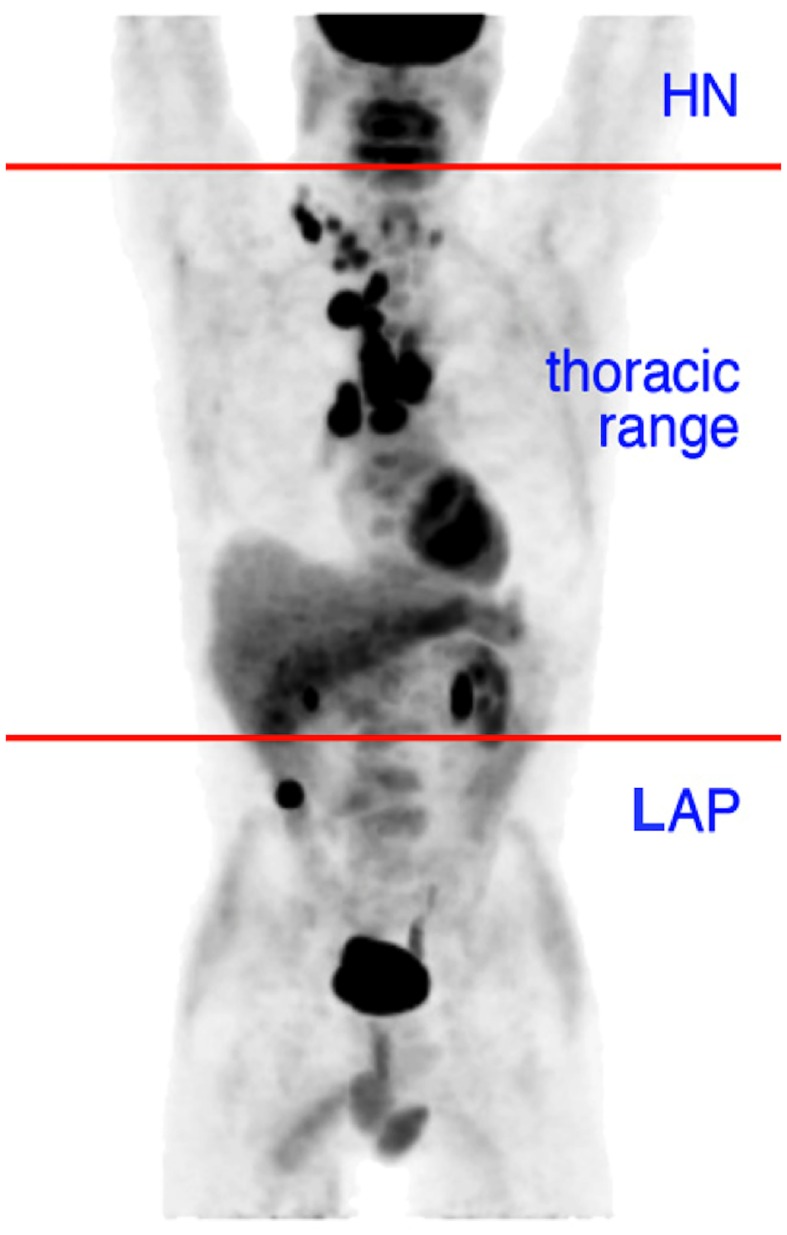
Scan regions. The regions evaluated in this study. The head and neck area (HN): all above the shoulder line. The lower abdominal and pelvic area (LAP): all below the caudal tip of the right liver lobe. The region between HN and LAP (chest and upper abdomen, thoracic range) is the region of interest for imaging of (suspected) lung cancer.

The nuclear medicine physicians (AIJA, JWAP, WMJS, AL, BWH, and WVV with 5, 2, 5, 1, 5, and 7 years of experience with FDG PET, respectively) independently and unblinded evaluated the existing reports of their own institution, and evaluated reported lesions. For each scan the presence and amount of lesions in three regions were documented. The head and neck area (HN): all above the shoulder line. The lower abdominal and pelvic area (LAP): all below the caudal tip of the right liver lobe. The region between HN and LAP (thoracic range) is the region of interest for imaging of (suspected) lung cancer (Fig 1).

### Image analysis

The PET/CT findings were evaluated for (1) the impact of detected lesions in HN and LAP in change on staging, (2) PET-driven additional diagnostic procedures and (3) the impact on patient management by analysis of medical files and/or consultation of the referring pulmonologist, reflecting the use of PET/CT in clinical practice.

Lesions reported on FDG-PET/CT that were considered clinically irrelevant (i.e. did not result in any clinical effect or analysis for the management of the initial index lung lesion within 3 months after performing the FDG-PET/CT scan) were considered “ignored” and thus not relevant in the process of the patients’ work-up and treatment for lung cancer. Lesion-based analyses were performed separately for the HN and LAP areas, and on a patient basis (for the combined HN-LAP areas).

## Results

The 5 participating centers included a total of 1059 patients (668 male and 381 female; average age 64 years, range 31–100 years). In 207 patients (19.7%), one or more FDG-avid lesions indicative of distant metastases or a second primary tumour in either the HN or LAP area were indicated in the report (Table 2).

**Table 2:** FDG PET-findings summarised by scan area.

Scan area	Patient based analysis of imaging results			Impact on lung cancer			Other findings	
	*suspect PET*	*Additional tests*	*Neglected / benign*	*Proven mets*	*Impact on stage*	*Impact on treatment*	*Confirmed benign disease*	*Confirmed second primary*
**HN**	7.2%	2.4%	3.3%	3.2%	0.5%	0.4%	0.7%	0%
**LAP**	15.8%	5.7%	4.1%	9.0%	0.8%	0.8%	2.7%	0.7%
**HN+LAP**	19.5%	7.8%	6.1%	10.0%	1.3%	1.2%	3.4%	0.7%

HN = head/neck area. AP = lower abdominal and pelvic area. HN+LAP = in either the HN or the LAP area. All listed percentages are relative to the total patient cohort.

**Fig 2:**
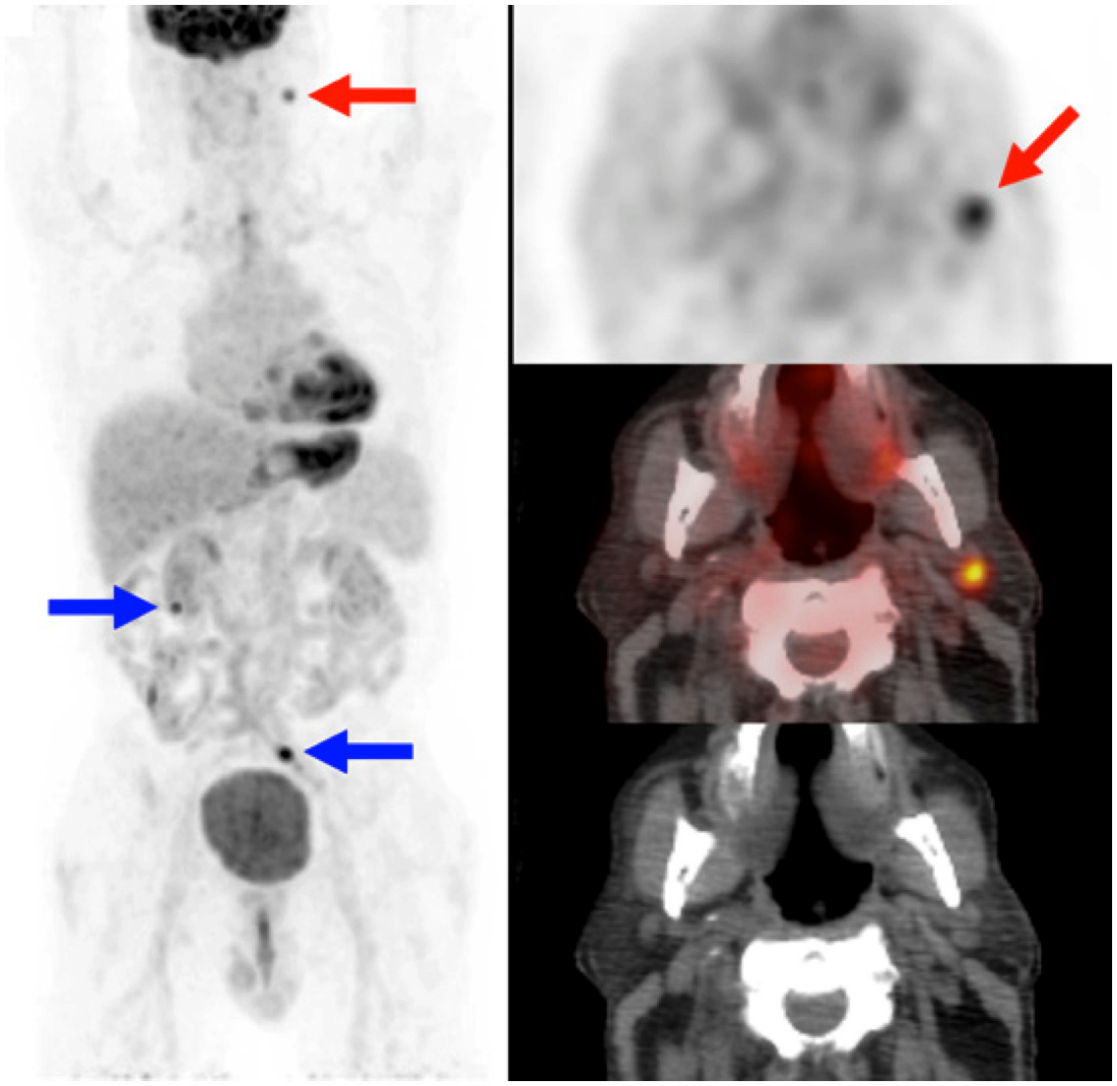
Example head/neck area. Significant false-positive finding on PET/CT in the head/neck area. Intense FDG uptake in a lymph node (red arrow), revealing inflammation in a patient who eventually did not have lung cancer. Bowel-hotspots were ignored (blue arrows). Left: maximum intensity projection of PET. Right: Corresponding transverse slices of PET (top), PET/CT (middle), and CT (bottom).

In the HN area, suspect FDG-avid lesions were identified in 7.2% of the patients. In 3.2% of patients metastatic disease from lung cancer in the HN area was considered based on PET, confirmed by additional investigations. This resulted in upstaging in only 0.5% and changed therapy (i.e. from surgery to chemotherapy, from chemotherapy with curative intend to chemotherapy with palliative intend) in 0.4%. Positive findings consisted of metastases in high cervical lymph nodes and the brain. Skeletal metastases were also detected in HN, but none of these upstaged the patient as they all had multiple distant metastases in the thoracic field of view. In addition, some Warthin tumours, benign thyroid nodules and benign lymph nodes were identified, but no proven second primary tumours in the HN area were detected (Fig 2). Altogether only one third of the suspected PET lesions was further evaluated. Nearly half of the lesions were considered clinically irrelevant or considered benign in the context of findings on the scan of a patient with a lung tumor.

**Fig 3:**
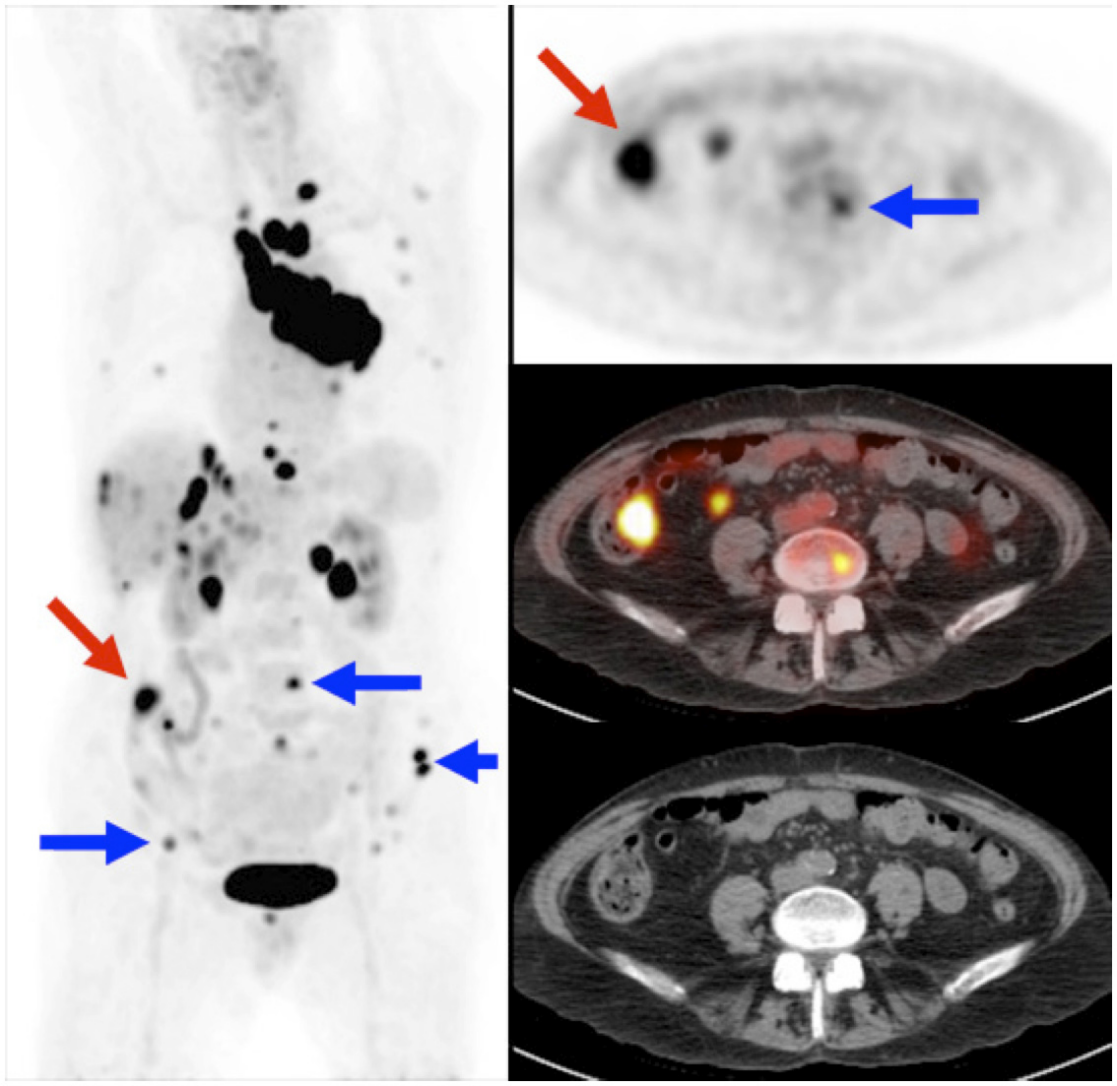
Example abdominal/pelvic area. True-positive but no-impact findings on PET/CT in the lower abdominal and pelvic area. Multiple skeletal metastases were detected (blue arrows), but these had no impact on staging as the thoracic field of view already showed multiple distant metastases (skeletal, adrenal glands, liver). An intense bowel hotspot (red arrow) is probably a colon carcinoma or large dysplastic polyp, but this was ignored as the prognosis of this patient was determined by the lung cancer. Imaging of the lower abdomen and pelvic area did not change the stage or therapy for this patient. Left: Coronal maximum intensity projection of PET. Right: Corresponding transverse slices of PET (top), PET/CT (middle), and CT (bottom).

In the lower abdomen/pelvic area, suspect FDG-avid lesions were identified in 15.8% of the patients. From all patients, 9.0% was considered to have metastatic disease from lung cancer in the LAP area based on PET, confirmed by additional investigations. This resulted in upstaging of only 0.8% of patients and changed therapy in 0.8%. Patients were upstaged due to metastases in the lumbar spine and pelvis, psoas muscle, adnex, subcutaneous tissue, and mesenterium. In addition, 4 clinically silent colon carcinomas, 1 bladder cancer, 1 schwannoma, and 1 unspecified abdominal malignancy were detected and subsequently proven (total 0.7% of patients), as well as numerous benign bowel polyps (Fig 3). Compared to HN, the LAP region showed more lesions, but had a similarly low impact on patient management for lung cancer.

In total, the classical “full field of view” (the whole body) FDG-PET/CT migrated stage in only 14 of 1059 patients (1.3%) due to the inclusion of the HN and LAP (for non-small cell lung cancer 3 patients from stage IIb to IV, 2 from IIIa to IV, 5 from IIIb to IV, and 2 from unspecified to IV, and for small cell lung cancer 2 patients from limited to extensive disease). The treatment of thirteen patients (1.2%) changed from curative to palliative intent; the remaining patient being upstaged was already considered for palliative treatment for other reasons. This indicates that when limiting the FDG-PET/CT scan field of view to ‘thoracic range’ results in correct staging in 98.7% of patients and identical management as full field of view PET/CT in 98.8% of patients.

There was no significant difference between institutes that included (in 718 patients) or omitted the brain (in 341 patients) (reported lesions in HN and LAP in 20.9% (24.3%-13%-25.4%) versus 19.3% (19.1%-19,6%), and impact on therapy 1.4% (1.6%-0%-2.7%) versus 1% (1.2%-0.7%)). The institute that performed whole body contrast-enhanced high dose CT in all patients (in a total of 264 patients versus the other 795 patients) reported more lesions (25.4%), but this did not result in more upstaging (1.1%).

Due to the additional FDG-PET/CT results in HN and LAP, a total of 96 additional investigations was ordered, with estimated total costs of €25,360 (Table 3). As only 14 patients were upstaged and another 7 patients were diagnosed with a second primary tumour, most of these investigations were performed for lesions that turned out to be benign or with little clinical relevance. The costs for additional investigations generated by FDG-PET/CT were relatively low for the entire population (€25,360 / 1059 = €24 per patient). The additional costs per patient upstaged or diagnosed with a second primary tumour were €1208 (€25,360 / 21).

**Table 3:** Additional investigations.

Additional investigations	Costs per unit (€)[19]	Number performed	Total cost (€)
Biopsy	50	18	900
Ultrasound	60	17	1,020
Bone scan	160	4	640
CT	200	4	800
MRI	200	21	4,200
Endoscopy	350	28	9,800
Operation	2,000	4	8,000
**Total**		**96**	**25,360**

The number of additional investigations performed for suspect FDG-avid lesions as seen on whole body FDG-PET/CT in the head/neck (HN) and lower abdomen/pelvis (LAP) areas of patients referred for evaluation of (suspected) lung cancer.

## Discussion

The added value of FGD-PET(/CT) imaging for characterization and staging of lung cancer is undisputed [20]. The procedure allows adequate non-invasive characterization of lung nodules, improves the accuracy of staging for lymph nodes and distant metastases, reduces the number of unnecessary invasive procedures such as mediastinoscopy and thoracotomy, allows better determination of prognosis, may improve therapy outcome, and is cost-effective [8,9,11,21–28]. Here, we have focused on the primary evaluation of lung nodules and primary staging of lung cancer in patients without a history of prior malignancy or symptoms of disease activity outside of the chest area. We chose for a multi-centre setting including a dedicated cancer clinic, a university hospital and general hospitals to evaluate the feasibility of a shorter scan field of view for PET/CT to avoid variations in locally applied imaging protocols for PET/CT. We showed that the high diagnostic accuracy is achieved in the ‘thoracic range’ area, while the FDG-PET findings of the HN and LAP areas have very limited clinical impact and may even result in unwanted delays and futile additional tests.

The total number of lesions suspect for malignancy outside the thoracic area was quite high (almost 20% of patients), but only a very limited fraction of these lesions had a clear clinical impact. Many findings were not of additional value even though there was clear suspicion or proof of metastatic disease, because imaging of the thoracic field of view had already established stage IV disease. This was notably true for skeletal metastases in the lumbar spine or cervical spine, which occurred relatively frequently (10%), but hardly ever without concurrent metastases in the thoracic spine or ribs (only in 0.2%).

A large part of the suspect PET-findings in HN and LAP was not further evaluated (almost one third of the described lesions), mainly because they were considered clinically irrelevant given the detected presence of (metastasized) lung cancer within the thoracic scanning field of view. From the evaluated lesions, many were found to be benign and irrelevant for staging after sometimes costly additional investigations (in 6.1% of patients, or nearly one third of the described lesions), and may have delayed the start of appropriate therapy for lung cancer. This reflects the balance between the very high sensitivity and relatively limited specificity of FDG-PET even when using an integrated PET/CT scanner, and the relatively low pre-test likelihood of metastatic disease from lung cancer in the HN and LAP regions [29]. Our results are in agreement with Marom et al., who previously reported that FDG-PET/CT for thoracic malignancies may reveal many irrelevant FDG-avid lesions outside the thorax, and Lardinois et al. who confirmed that about half of such lesions are indeed benign or clinically irrelevant [30,31].

Second primary malignancies occur relatively frequently in patients with lung cancer, and the ability of FDG-PET to detect these is well known [18]. Treating lung cancer while another malignancy remains undetected can be considered oncologically undesirable, and for this reason many consider whole body PET essential. Our series featured lesions suspected of possible second malignancies in several cases, of which only a small part was actually considered oncologically relevant (0.7%). This number is remarkably similar to the detection rate of cancer (0.97%) reported by Minamimoto et al, using PET/CT in the cancer screening study in 50558 patients in Japan [32]. Gutman et al. also found that only a few of the most intense hotspots of a large number of bowel hotspots represented second primary malignancies [33]. Thus, second primaries may be detected by FDG-PET, but in many cases these are clinically irrelevant in the presence of (metastasized) lung cancer, while requiring quite a number of additional investigations per positive result. Although not specifically addressed in our retrospective analysis, it can be imagined that false-positive or clinically irrelevant FDG-PET findings may contribute to prolonged patient stress in the diagnostic period prior to the start of therapy, e.g. due to delays or physical discomfort due to additional invasive procedures (e.g. colonoscopy, biopsy). A possibly negative effect of a diagnostic delay of up to several weeks on the final oncological outcome of lung cancer is difficult to assess, but may occur in a (limited) number of cases.

Using stand-alone FDG-PET, Reed et al found unexpected distant metastases in 5.2% of patients with lung cancer who were initially considered operable [23]. However, these relevant metastases may not be distributed evenly in the body. Acquino et al demonstrated that thoracic field of view FDG-PET has sufficient value, and that extending to wholebody FDG-PET hardly has any impact on staging of lung cancer although it detects more metastases [34]. Our findings confirm that the same hold true for integrated PET/CT, although some methodological differences exist. We did not exclude small cell lung cancer or non-confirmed PET findings, in order to reflect daily clinical practice without selection bias. The use of integrated PET/CT instead of a separate PET most likely excluded several false positive findings [35]. We feel these results represent the current situation in most diagnostic centers.

Some clinicians regard the FDG-PET/CT as the most efficient imaging modality and would rather use it as a “one-stop-shop” and achieve as much information as possible in one session via whole body imaging in all patients. However, we believe the application of any imaging protocol needs to be tailored to clinical demands. Customizing PET imaging protocols to specific clinical questions has been suggested for many situations. Examples are extension of the scanning field of view to a full head-to-toe in melanoma and multiple myeloma or a very limited scan field of view with optimized acquisition and reconstruction parameters in the head and neck area [36,37]. Other examples of customized protocols include delayed imaging of e.g. brain tumours or dual time point imaging for discrimination of tumour and inflammation [38,39].

## Conclusion

We conclude that staging of newly diagnosed or suspected lung cancer with FDG-PET/CT is very accurate when evaluating the thorax and upper abdomen. Expanding the field of view to include the head and neck, and lower abdominal and pelvic field of view may reveal additional information, but also induces oncologically irrelevant additional investigations and delays with very limited impact on patient management. Therefore, additional positive findings outside the chest and upper abdomen should be reported with extreme caution.
